# Septic Arthritis of the Sternoclavicular Joint

**DOI:** 10.5811/cpcem.1563

**Published:** 2024-01-25

**Authors:** George V. Koshy, Richard Davis, Christopher Wilson

**Affiliations:** Department of Emergency Medicine, Geisinger Medical Center, Danville, Pennsylvania

**Keywords:** *septic arthritis*, *sternoclavicular joint*, *ultrasound*

## Abstract

**Introduction:**

Sternoclavicular joint (SCJ) septic arthritis is a rare but rapidly fatal joint infection. Without proper medical or surgical management, it can progress to osteomyelitis, chest wall abscess, mediastinitis, or myositis.

**Case Report:**

A 57-year-old male with a past history of intravenous drug use presented to the emergency department (ED) with chest tenderness of one week duration. Vital signs were unremarkable, and exam was notable for tender, raised right SCJ without any fluctuance. On point-of-care ultrasound we noted fluid collection and capsular distention along the SCJ, which aided in rapidly diagnosing septic arthritis. The patient was immediately started on antibiotics and taken to the operating room for excision and debridement.

**Conclusion:**

While computed tomography is routinely used in the emergency department for diagnosing septic arthritis, ultrasound offers a rapid and safe alternative for diagnosis.

Population Health Research Capsule What do we already know about this clinical entity?

*Sternoclavicular joint (SCJ) septic arthritis is a rare but serious condition, constituting less than 1% of bone and joint infections.*

What makes this presentation of disease reportable?

*In our case, ultrasound revealed intra-articular effusion and capsular distention, which suggested presence of septic arthritis and possible osteomyelitis.*

What is the major learning point?

*Given that >90% of cases do not have joint swelling and symptoms are vague, the clinician should have a high index of suspicion for SCJ septic arthritis.*

How might this improve emergency medicine practice?

*Point-of-care ultrasound may be used in the initial diagnostic consideration when advanced imaging or orthopedic consult is not available.*

## INTRODUCTION

Sternoclavicular joint (SCJ) septic arthritis is a rare but potentially fatal condition, representing less than 1% of all bone and joint infections.^1^ It often presents insidiously with vague and poorly localized signs. The majority of the laboratory markers and imaging modalities are nonspecific with definitive diagnosis being arthrocentesis with cell count and culture. It is classically seen in males with a mean age of 45 years and associated with intravenous (IV) drug use, diabetes, and infections in other locations. However, more than 25% of patients have no risk factors.^2^ ^,^ ^3^ Without proper medical or surgical management, it can progress to osteomyelitis, chest wall abscess, mediastinitis or myositis.^4^ ^,^ ^5^ Thus, a high index of suspicion with prompt recognition and management in the emergency department (ED) is vital to avoid poor outcomes. Here we present a case of SCJ septic arthritis with associated osteomyelitis and acute myositis, with rapid assessment and diagnosis using ultrasound.

## CASE REPORT

A 57-year-old male with a past medical history of IV methamphetamine abuse, hepatitis C, and hypertension presented to the ED for tenderness along the right anterior chest. His symptoms started about a week prior to presentation with an audible pop and subsequent pain that he felt in his right sternoclavicular area. Symptoms had been progressively worsening in severity, without radiation, and no aggravating or alleviating factors. He tried taking acetaminophen and ibuprofen without much relief. He denied any associated symptoms including shortness of breath, fevers, or nausea and vomiting. He also denied history of direct injection to this joint or any other type of direct trauma. Social history was notable for homelessness, poor medication compliance, marijuana use, and a history of IV substance use 10 years prior, but he denied recent drug or alcohol use.

**Image 1.**
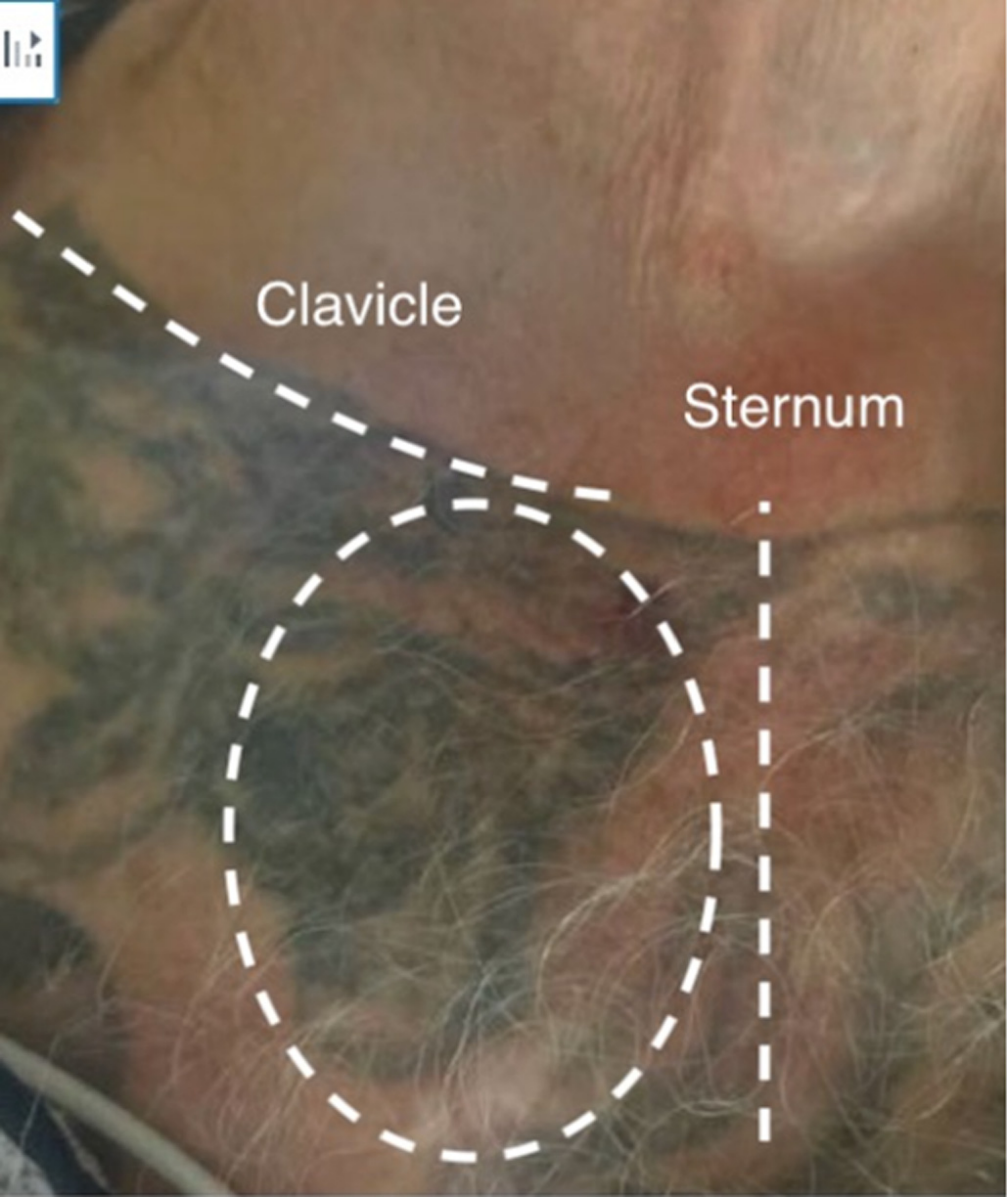
Sternoclavicular joint on initial presentation. No fluctuance noted.

**Image 2.**
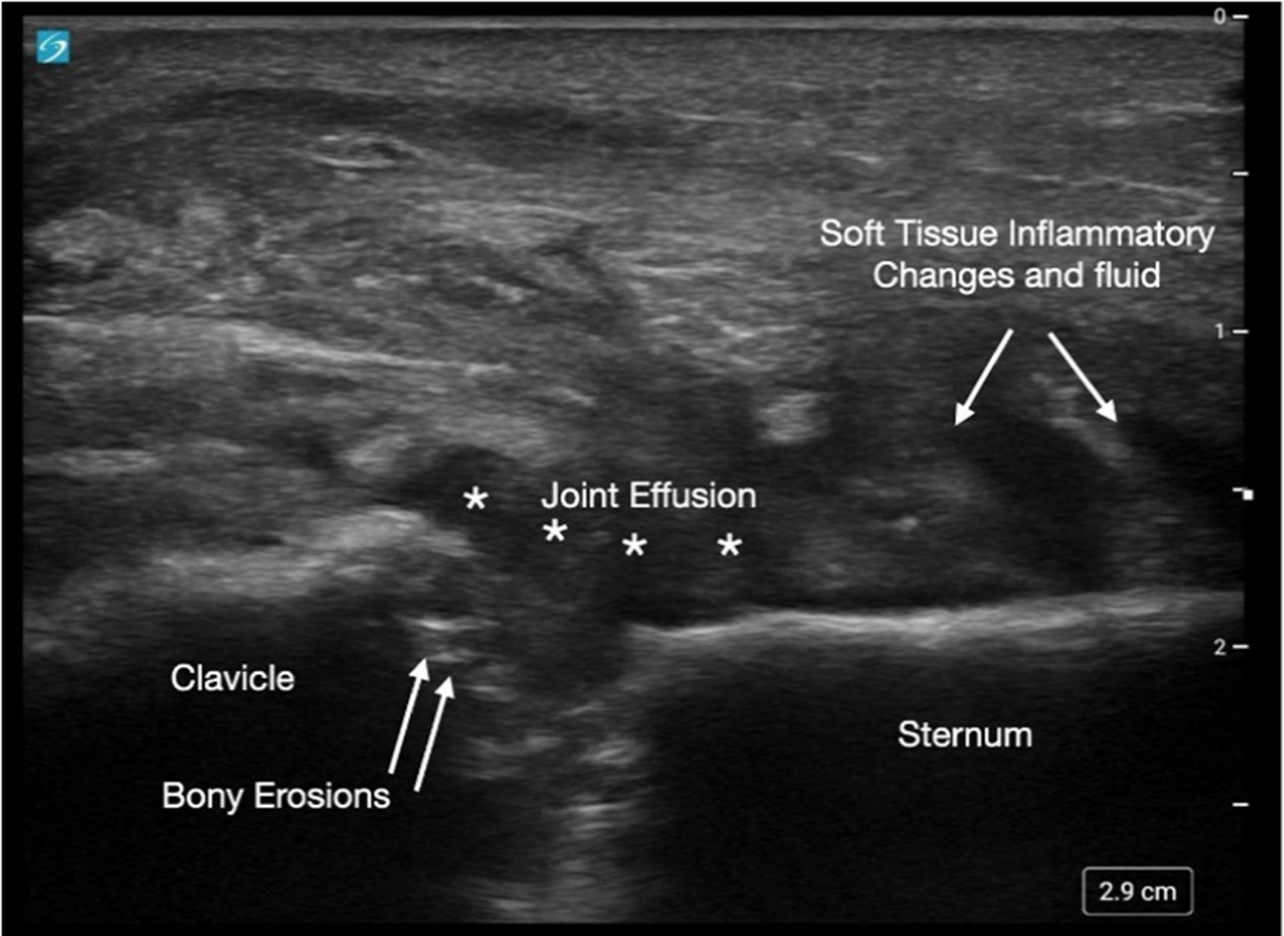
Point-of-care ultrasound of the sternoclavicular joint revealing intra-articular effusion and capsular distention.

Upon presentation, vital signs were notable for temperature of 97.2° Fahrenheit, blood pressure 128/76 millimeters (mm) of mercury, heart rate 87 beats per minute, respiratory rate 16 breaths per minute, and room air oxygen saturation of 98%. Physical examination was notable for tender, raised right SCJ without any fluctuance, as seen in Image 1. Labs were notable for white blood cell count of 12.3 × 10^9^/liter (L) (reference range 4.5 to 11.0 × 10^9^/L); platelets of 639 × 10^9^/L (150 to 400 × 10^9^/L); erythrocyte sedimentation rate of 120 mm/hour (hr) (0 to 15 mm/hr), and C-reactive protein of 34 milligrams (mg)/L (8–10 mg/L). A rapid point-of-care ultrasound revealed a fluid collection and capsular distention along the SCJ (Image 2) concerning for septic arthritis. Orthopedics was consulted.

**Image 3.**
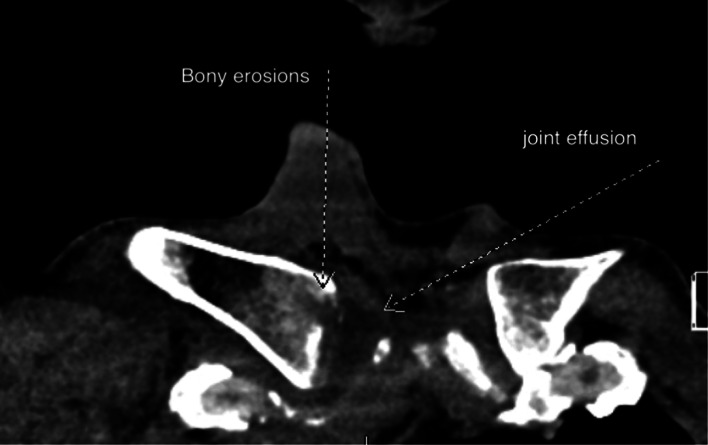
Computed tomography revealing joint effusion, bony erosions in the clavicle and adjacent sternal manubrium.

While waiting for orthopedics evaluation, computed tomography (CT) was performed for confirmation, which revealed SCJ effusion, bony erosions in the clavicle and adjacent sternal manubrium, and associated extensive soft tissue inflammation, concerning for septic arthritis and osteomyelitis (Image 3). Orthopedics was unable to aspirate the joint and, thus, interventional radiology was consulted for joint aspiration and biopsy. Due to absence of septic signs, the patient was not immediately started on antibiotics. Post biopsy, the patient was started on piperacillin/tazobactam and admitted for further management. Joint aspirate cultures were positive for Gram positive cocci in chains, and antibiotics was changed to ceftriaxone as per infectious disease recommendation. The patient was subsequently taken to the operating room for right medial clavicle excision and SCJ debridement and irrigation. He remained afebrile in the post-operative period. Tissue cultures were positive for *Streptococcus pneumoniae*. He was discharged within a week with outpatient follow-up with infectious disease and orthopedics.

## DISCUSSION

Infections in the SCJ carries a high risk of systemic complications. Joint effusions in this joint progress slowly, with large effusions associated with infiltration into the systemic circulation. The most common symptoms on presentation include fever, chest pain, and neck pain.^6^ A review by Ross et al^7^ found that up to 96% of cases do not have swelling of the joint. Given the relatively vague presentation, and lack of systemic complaints, it is important that the emergency clinician carry a high index of suspicion especially for patients with known risk factors.

Traditionally, CT is the most commonly used tool followed by magnetic resonance imaging (MRI) in making the diagnosis. However, there is little data in the literature about using ultrasound to detect SCJ septic arthritis. In a case report by Kawashiri et al, ultrasound of a SCJ septic arthritis revealed inflammation and appearance of bony erosions, which aided in rapid assessment and diagnosis. In a case report by Monteiro et al,^9^ use of ultrasound aided in the early assessment of power Doppler-positive synovitis of the SCJ. Findings of joint effusion and irregularities of bony margins, which appear as anechoic or complex non-septate collections are suspicious of infections. In our case, ultrasound revealed intra-articular effusion and capsular distention, which suggested presence of septic arthritis and possible osteomyelitis (Image 2).

While we used confirmatory CT, we had consulted orthopedics for suspected septic arthritis based only on ultrasound findings. This is especially significant for EDs where CT or MRI are not readily available and diagnostic evaluation can be initiated based on ultrasound alone. Some emergency physicians practice in facilities where advanced diagnostic imaging and orthopedic consultation may not be immediately available and necessitate transfer of the patient for further diagnostic and definitive care. While confirmatory studies such as CT and MRI have been traditionally used in diagnosing SCJ septic arthritis, we hope that this paper adds to the evidence that point-of-care ultrasound may be used in the initial diagnostic consideration.

Management of SCJ septic arthritis includes surgical debridement along with IV antibiotics. Most common etiologic agents include *Staphylococcus aureus* (58%), methicillin resistant *staphylococcus aureus* (5%), *Pseudomonas aeruginosa* (10%), and *Brucella melitensis* (7%).^6^ ^,^ ^8^ Once blood cultures are drawn and patient meets sepsis criteria including fever or tachycardia, initial management with broad spectrum antibiotics such as cefazolin or piperacillin/tazobactam is recommended.^5^ ^,^ ^10^

## CONCLUSION

Septic arthritis of the sternoclavicular joint is an uncommon entity among joint infections, without a specific defined diagnostic or therapeutic pathway. Ultrasound offers a rapid and safe alternative for diagnosis. Prompt recognition and management with antibiotics and early involvement of multidisciplinary team can lead to improved patient care and outcomes.
